# A method for estimating spikelet number per panicle: Integrating image analysis and a 5-point calibration model

**DOI:** 10.1038/srep16241

**Published:** 2015-11-06

**Authors:** Sanqin Zhao, Jiabing Gu, Youyong Zhao, Muhammad Hassan, Yinian Li, Weimin Ding

**Affiliations:** 1 College of Engineering, Nanjing Agricultural University, 210031, Nanjing, Jiangsu, China; 2 Engineering Laboratory of Modern Facility Agricultural Technology and Equipment in Jiangsu Province, 210031, Nanjing, Jiangsu, China

## Abstract

Spikelet number per panicle (SNPP) is one of the most important yield components used to estimate rice yields. The use of high-throughput quantitative image analysis methods for understanding the diversity of the panicle has increased rapidly. However, it is difficult to simultaneously extract panicle branch and spikelet/grain information from images at the same resolution due to the different scales of these traits. To use a lower resolution and meet the accuracy requirement, we proposed an interdisciplinary method that integrated image analysis and a 5-point calibration model to rapidly estimate SNPP. First, a linear relationship model between the total length of the primary branch (TLPB) and the SNPP was established based on the physiological characteristics of the panicle. Second, the TLPB and area (the primary branch region) traits were rapidly extracted by developing image analysis algorithm. Finally, a 5-point calibration method was adopted to improve the universality of the model. The number of panicle samples that the error of the SNPP estimates was less than 10% was greater than 90% by the proposed method. The estimation accuracy was consistent with the accuracy determined using manual measurements. The proposed method uses available concepts and techniques for automated estimations of rice yield information.

Rice (*Oryza sativa*) is a primary cereal crop that is consumed by more than half of the world’s population, and rice is particularly important in China because of the extensive population. Therefore, accurate yield estimates are extremely important for ensuring the safety of rice production and providing a continuous supply. Many methods for estimating rice yields have been proposed and are currently in use, including methods that use large-scale full coverage and regional-scale sampling surveys. Large-scale yield estimation is a promising method because of the adoption of remote sensing and satellite technologies, which obtain timely and objective yield-related traits, such as LAI, NDVI, fPAR and NPP^1^,^2^, and therefore provide a prediction of the entire yield. Unfortunately, the large-scale yield estimation method is not suitable for organizations other than research institutions and state departments because of the high costs. By contrast, regional-scale rice yield estimation uses a traditional statistical sampling method, which is more flexible and has higher accuracy than the large-scale yield estimation method, particularly for measuring the panicle traits and estimating the yields of small plots. Hence, the regional-scale yield estimation method is typically adopted and is widely approved for use in studies of high-yield breeding.

The regional-scale yield estimation method comprises four yield components, which include the panicle number per plant, spikelet number per panicle (SNPP), filling rate and 1000 grains weight. Manual low-throughput measurement methods for determining these traits are time consuming, and the results are unreliable. Moreover, the SNPP is the most difficult to quantify manually, particularly when the SNPP is greater than 200. Thus, there is an urgent demand for an automated method for rapidly estimating the SNPP.

With the rapid development of optical imaging techniques and computer technology, image analysis has become an effective method for the automated measurement of rice panicle traits, including analysis using machine-vision-based facilities^3^,^4^,^5^,^6^,^7^ and 2-D image-based panicle phenotyping software^8^,^9^,^10^. Among these image analysis methods, machine-vision-based facilities can measure traits efficiently but are very expensive and large, and therefore, these facilities are not available for field measurements in real time. Special 2-D image-based panicle phenotyping software (e.g., PASTAR/PASTA Viewer^8^, P-TRAP^9^ and PANorama^10^) is cost-effective, although this methodology is inefficient because each spikelet/grain on a panicle must be spread out manually before the panicle image is captured.

The size scales of the spikelet/grains and panicle branches are significantly different, and therefore, it is difficult to simultaneously extract both panicle branch and SNPP information from images having the same resolution. Existing image analysis methods do not simultaneously consider the accuracy and efficiency. Moreover, image resolution is the key factor that affects the accuracy and the efficiency. To use a lower resolution and meet accuracy requirements, expert knowledge has been adopted to analyse the relationships between the different panicle phenotypes, and the results have been used to match the different resolutions^11^. Fortunately, relationships have been identified between the SNPPs and the branching components (e.g., number, order and length of branches). Thus, a reasonable and novel relationship model between the panicle branches and the SNPPs was developed to match the resolution of the images.

The parameters of the relationship models have been adjusted for changes in rice variety, growth environment and climate features. Moreover, serious difficulties have been encountered in the establishment of a universal model. However, the parameter calibration has been used as an engineering method for parameter determination to ensure model universality. Consequently, the objectives of this study were to integrate interdisciplinary advantages, such as the use of a biological perspective, a relationship model to solve the resolution problem, and an engineering perspective to adopt the calibration of the parameters to solve the problem of model universality. The contents of this report are as follow: (1) a relationship model was established between the panicle branches and the SNPPs to match the resolution, (2) an image-processing algorithm was designed to rapidly obtain the characteristics of panicle branches, and (3) a 5-point calibration method was proposed and applied to rapidly determine the model parameters.

## Methods

### Rice panicle collection

In this experiment, 1100 panicles were analysed. The characteristics of all panicles are listed in Table 1, including the variety, collection date, sample number, cultivar and symbol. The mature panicles of each rice variety were cut from an area of approximately 0.5 m^2^. The panicles (A, B, C and E) of 6 rice varieties were collected from the Zhujiang experimental farm at the Nanjing Agricultural University, and the other panicles (D and F) were collected from fields in Jinhu County, Jiangsu Province, China. After collection, the panicles were labelled, spread on paper, and allowed to air-dry for 2 weeks in the laboratory.

### Establishing a relationship model among traits between the panicle branches and the SNPPs

#### Manual measurements of the panicle traits

**Figure 1:**
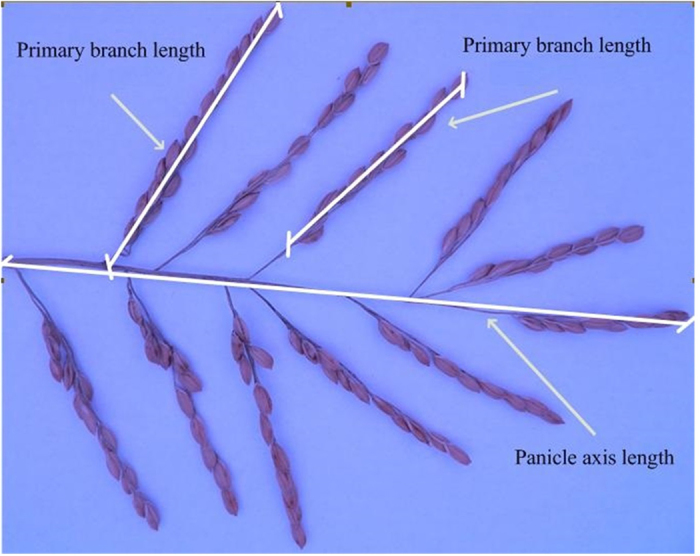
Definitions and measurement method for the lengths of the primary branch and the panicle axis used in this study, designated as PBL1, PBL2 and PAL. All primary branch lengths obtained using methods 1 or 2 were summed as the total length of the primary branch, designated as TLPB1 and TLPB2.

**Figure 2:**
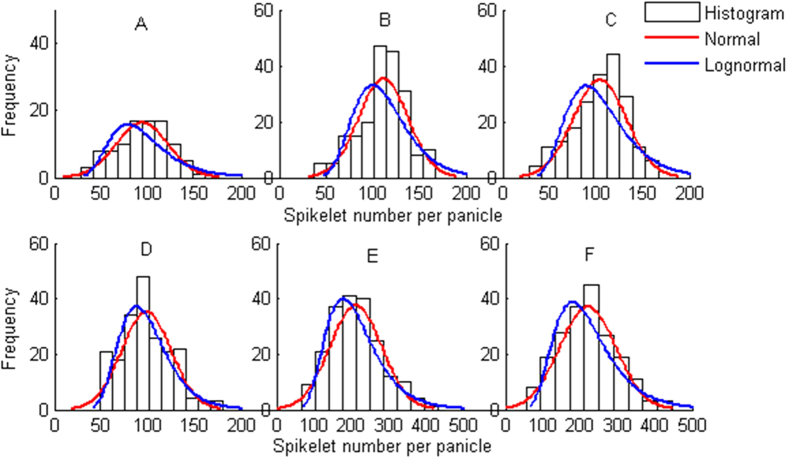
Normal and lognormal distribution histograms of the spikelet number per panicle (SNPP) for 6 rice varieties.

A rice panicle was first manually spread out (Fig. 1), and the panicle axis length (PAL) and primary branch length (PBL) were marked with white lines. The PBL was measured using two methods that are dependent on the starting position. Therefore, the PBLs were recorded as PBL1 and PBL2. All of the PBLs on a panicle were summed to obtain the total length of the primary branch (TLPB1 and TLPB2). All lengths were measured using a centimetre ruler, and the SNPPs were manually counted. Furthermore, normal and lognormal histograms of the SNPPs were constructed to explain the statistical significance of the collected panicle samples (Fig. 2).

#### Modelling the relationships among traits

**Figure 3:**
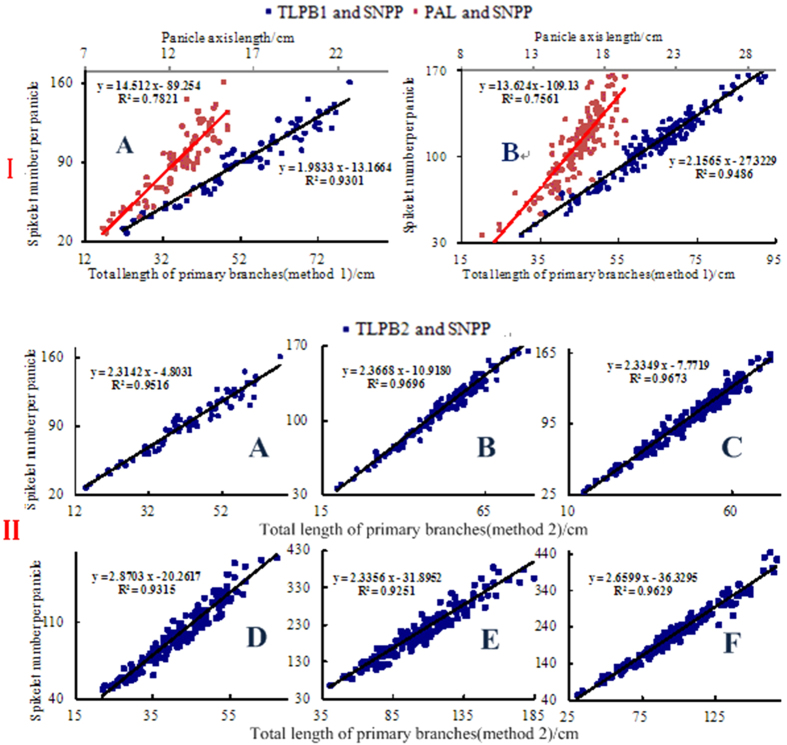
Relationships between the traits and the spikelet number per panicle.

To compare the correlations between the length traits (PAL, TLPB) and the SNPPs, 300 panicles (A and B, see Table 1) were first measured. Eighty percent of the trait data were then randomly chosen for the scatter plots for each variety. Linear regression equations were fitted based on the scatter plots, and the coefficient of determination *R*^*2*^ was calculated using EXCEL 2003 software (Fig. 3). The coefficient of determination, *R*^*2*^, showed that TLPB was more strongly correlated with SNPP than with PAL, and the correlation of TLPB2 with SNPP was stronger than that with TLPB1. TLPB2 showed a stronger correlation with the positions of the SNPPs than did TLPB1. Consequently, the remaining panicles were used to validate the stability of the relationship model between TLPB2 and SNPP (Fig. 3 II B, C, D and E). Thus, SNPP was proportional to TLPB2, and the proposed relationship model was stable for all experimental varieties. SNPP counts were converted to measure TLPB2. This linear model was used to calculate the SNPPs based on TLPB2. Thus, the resolutions of the panicle branches and the spikelets/grains were matched.

### Developing an image-processing algorithm to rapidly obtain the characteristics of panicle branches

#### Image acquisition

**Figure 4:**
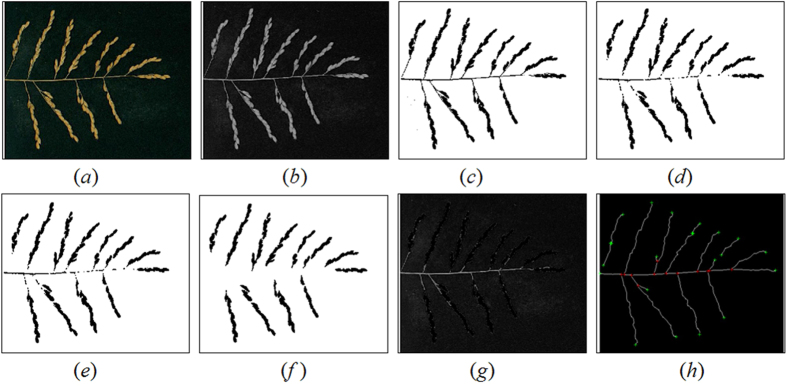
The steps in the image-processing used to extract the region of the primary branches defined by method 2. (***a***) RGB image of spread out panicle. (***b***) Grey scale image. (***c***) Binary image. (***d***) The image after applying the morphological opening with a 1 × 1 disk. (***e***) Image with holes filled. (***f***) The image with small objects removed. (***g***) The extraction results represented as (***b*****–*****b***)*(-*f*). (***h***) The panicle skeleton and the vertices. Junctions (red + ), Terminals (green + ).

To increase the speed of the automated TLPB2 measurement compared with manual measurements, an image analysis program (see Appendix) was designed using MATLAB 2013a (The MathWorks, Inc., Natick, Mass). First, a rice panicle was spread out and scanned to acquire the image (72 dpi, RGB colour; Fig. 4a) with a scanner (MICROTEK, ScanMaker E900).

#### Extracting the primary branch region

The subsequent image analysis steps for extracting the PBL2 were as follows: the raw RGB image (Fig. 4a) was converted to grey scale (Fig. 4b) using the (R + G + B)/3 component; a binary image (Fig. 4c) was obtained using the Otsu automatic threshold method^12^; to obtain the region of the PBL2 (attached to grain), a morphological opening operation was used to remove only the thin parts of the panicle and leave the spikelets on the branches intact with a 1 × 1 disk-structuring element (Fig. 4d); the holes were filled (Fig. 4e) using a morphological reconstruction algorithm^13^; all of the connected components with fewer than 100 pixels were removed; and the regions of the PBL2 were extracted (Fig. 4f). The equation (*b*–b*(-*f*)) was used to observe the extraction results (Fig. 4g). In each step of the extraction, the functions used included ‘rgb2gray’, ‘im2bw’, ‘imopen’, ‘imfill’, and ‘bwareaopen’.

#### Calculating the TLPB2 and area traits

Furthermore, TLPB2 was calculated from the region (black parts) in Fig. 4f. PBL2 was defined as the major axis of the ellipse that had the identical normalized second central moments as the region^14^. All of the primary axes (PBL2) on a panicle were summed to obtain TLPB2. More importantly, the region area trait was also calculated, which was equal to the actual number of pixels belonging to the region. Thus, the area trait also corresponded with the SNPP. In this study, the ‘regionprops’ function was used to calculate TLPB2 and the area.

#### Verifying accuracy and robustness of the image-processing algorithm

**Figure 5:**
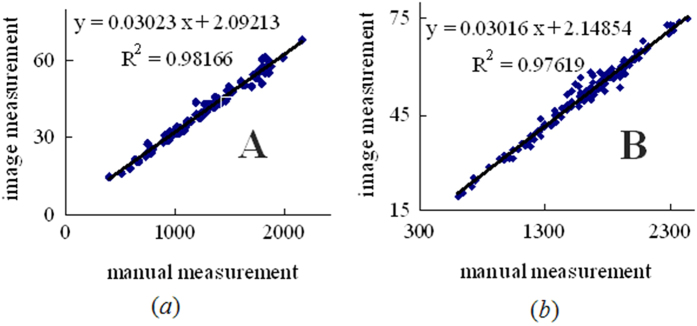
Comparison of the manual and image-based measurements of the total length of the primary branches.

To assess the accuracy and repeatability of the proposed algorithm, 300 panicle images (A and B) were initially processed. Additionally, TLPB2 was verified using a comparison of the coefficient of determination *R*^*2*^ between the image-based measurements and the manual measurements (Fig. 5). The image-based measurements were in strong agreement with the manual measurements. Thus, the proposed image-processing program had excellent repeatability and was robust. Finally, all of the remaining panicle images were processed to obtain the TLPB2 and area traits.

#### Establishing the relationship model between image traits and SNPPs with the adoption of 80% of samples

**Figure 6:**
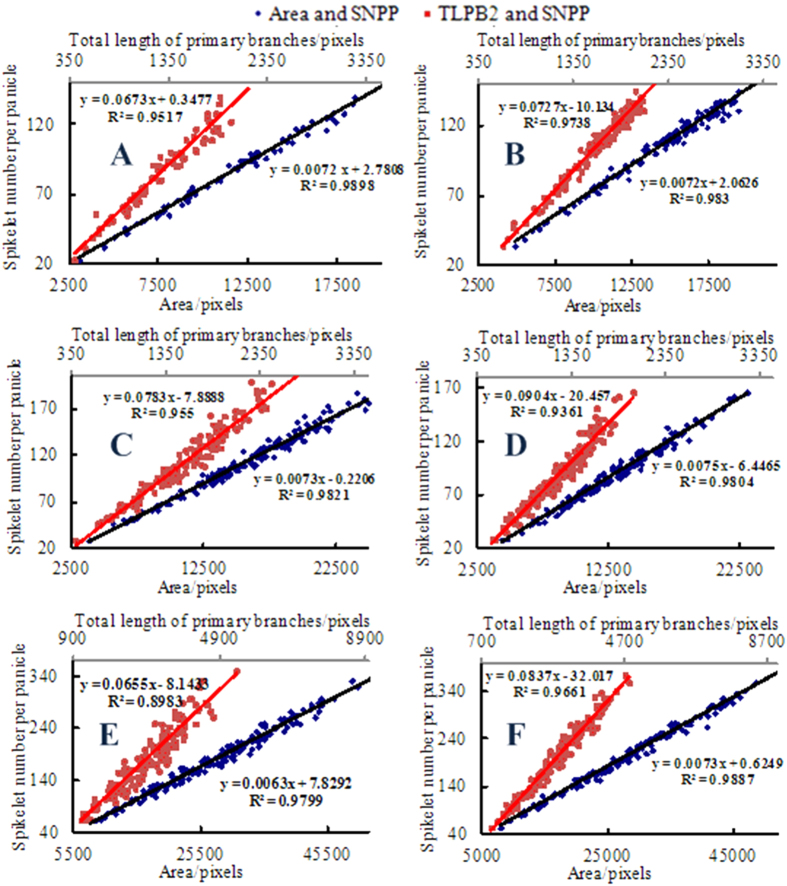
Relationships between the panicle traits obtained from the image analysis method and the spikelet numbers per panicle for 6 rice varieties.

Similarly, to establish the correlations between the traits (area, TLPB2) and the SNPPs, eighty percent of the trait data were randomly selected to construct scatter plots for each variety. The linear regression equations were fitted and the coefficient of determination *R*^*2*^ was calculated using EXCEL 2003 software (Fig. 6). Based on the coefficient of determination *R*^*2*^, the area and TLPB2 were significantly correlated with the SNPPs. Hence, the linear relationship models between the image traits and the SNPPs were exploited to measure the experimental samples.

#### Use of a 5-point calibration model to rapidly determine the model parameters

**Figure 7:**
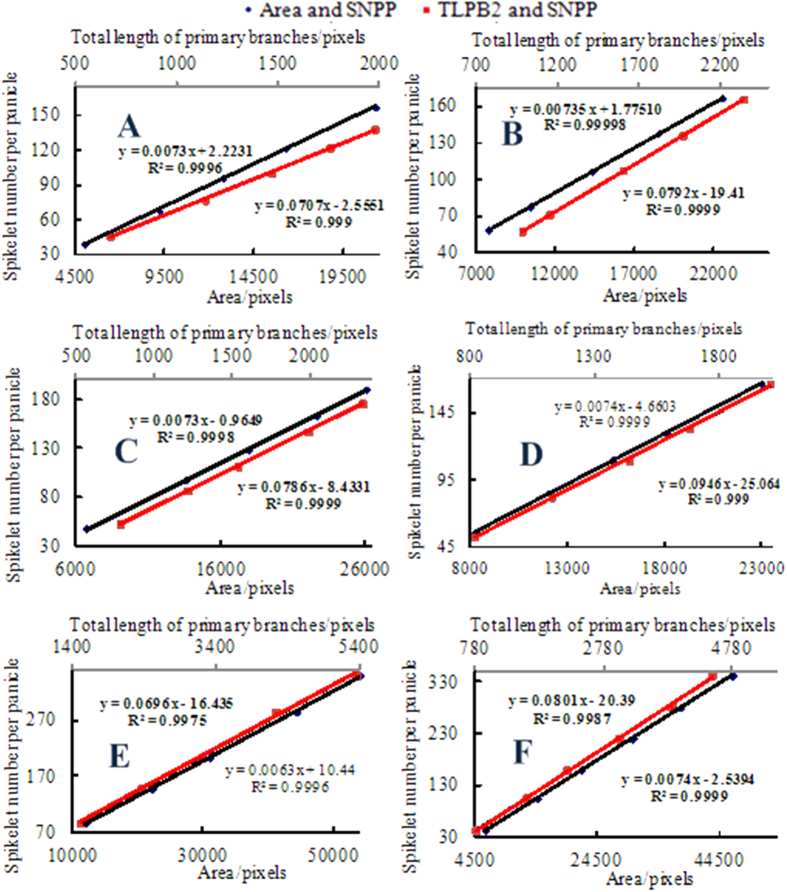
Establishment of the calibration curves between the panicle traits obtained from the image-based measurement method and the spikelet numbers per panicle for 6 rice varieties.

The SNPP was linearly proportional to the image traits (TLPB2, area), and this relationship was universal for each experimental variety. The key to rapidly estimating the SNPP was that the linear regression equation was determined quickly. In other words, the slope and intercept must be determined rapidly. Thus, the 5-point calibration models were used according to the linear relationship, noted as calibration curves. Importantly, the 5 samples must be chosen uniformly throughout the entire scale (Fig. 7). As expected, the high coefficient of determination *R*^2^ of the calibration curves indicated a better linear relationship.

## Results

### Accuracy assessment of the proposed estimation method

To assess the accuracy of estimations using the proposed method, the estimation error was calculated according to formula 1. Among the components of the formula, *SNPP* (*manual*) is the number counted manually, and *SNPP* (*cal*) is the number calculated using the respective linear regression equations, including those from Figs 3II, 6 and 7. The panicle samples used to establish the model were not used to calculate the estimation error.


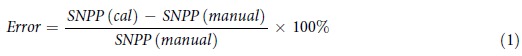


The estimation error statistics are listed in Table 2. The number of panicle samples that the error of the SNPP estimates was less than 10% was greater than 90% in the use of area models. The area trait was the most accurate trait for the estimation of SNPP. Moreover, the area estimation results were equivalent regardless of whether the eighty percent of the trait data model or the 5-point calibration model was used. However, the 5-point calibration model was much faster than the eighty percent of the traits data model. Hence, the proposed method of integrating the image-analysis and 5-point calibration model was effective in rapidly estimating the SNPP.

The error of using the length trait (TLPB2) to estimate SNPP was more than that of the area trait, and the order of error in the use of the TLPB2 trait was manual <image (20% test) <5-point calibration test. This order occurred because the primary branches could not be straightened when the panicles were scanned, which led to a low accuracy of the TLPB2 in the imaging measurement. However, this low accuracy had almost no effect on the area measurement. Moreover, the adhesion of the primary branches also had no effect on the area calculation, whereas this adhesion could affect the TLPB2 calculation using imaging. Additionally, the area trait could incorporate the grain size, whereas the TLPB2 could encompass only the grain length. Consequently, the area measurement was not only more accurate but was also much easier to use than the TLPB2 method. Notably, the underlying assumption of the proposed estimation method is that the grain size and the grain filling percentage are approximately uniform. Therefore, the panicle samples for each variety were preferably collected from the same location to ensure the highest accuracy of the estimation.

### Model application for estimating the spikelet number per square (SNPS)

The SNPS is a primary target in rice yield assessments, and the SNPS is calculated by averaging the SNPPs. The estimation error was calculated using formula (2). Among the components of the equation, the *TRUE* value was obtained from the average of the SNPPs that were counted manually. The *SNPS (cal)* was calculated by averaging the SNPPs estimated by the corresponding linear regression equations. The panicle samples for the modelling were not used to calculate the deviation.


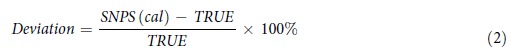


The deviation statistics are listed in Table 3. The SNPS estimation was close to the actual value, regardless of whether the TLPB2 calibration model or the area calibration model was adopted. The estimated deviation of the SNPS was clearly less than 5%, which was acceptable. The SNPS could be calculated exactly using the proposed method.

## Discussion

A rapid method for estimating the SNPP was proposed that integrated image analysis and a 5-point calibration model. The 5-point area calibration model was most accurate for the estimation of the SNPP. Additionally, the SNPS was precisely calculated, regardless of whether the 5-point area calibration model or the 5-point TLPB2 calibration model was used. The proposed method will be a useful tool for analysing the rice panicle phenotype in various areas of rice production, such as the estimation of field production and super-high-yield breeding programs.

In contrast to existing image-analysis methods, including PASTAR/PASTA Viewer, P-TRAP and PANorama^8^,^9^,^10^, which required spreading out each spikelet/grain preparation as well as a high-resolution panicle image, with the proposed method, it was necessary only to spread out each primary branch to obtain a lower-resolution image (72 dpi). The accuracy and efficiency were both accounted for simultaneously with the adoption of the proposed method, particularly for the simultaneous analysis of panicle traits and SNPP using a lower-resolution image. The panicle skeleton and the vertices were extracted using the thinning and look-up table algorithms^15^, and the results are shown in Fig. 4h.

The resolution was the key factor that affected the accuracy and efficiency of the image-analysis methods. For the image analysis, a substantial limitation has been the non-destructive measurement of characteristics at different scales with the same resolution. However, using the panicle physiological characteristics to model the relationships between traits such as the TLPB and the SNPP was the most promising means to solve the problem of resolution at different scales. Additionally, the parameters of the relationship models can vary with changes in the variety, climate, and growth environment, among other factors. Therefore, the establishment of a universal model was a serious problem; however, parameter calibration using an engineering method to calibrate the parameters was employed to solve the problem of developing a universal model. Consequently, the complementary advantages of the interdisciplinary approach were essential for simultaneously accounting for the accuracy and efficiency. This approach provided a useful tool for further investigating the interactions among the genes and the environment for the rice panicle.

## Additional Information

**How to cite this article**: Zhao, S. *et al.* A method for estimating spikelet number per panicle: Integrating image analysis and a 5-point calibration model. *Sci. Rep.* **5**, 16241; doi: 10.1038/srep16241 (2015).

## Figures and Tables

**Table 1:** Details regarding the rice panicles used in this study.

Rice variety	Collection date	Sample number	Cultivar	Symbol
Wuyijing	2011.10.25	100	japonica	A
Wuyuejing No. 21	2012.10.25	200	japonica	B
Nanjing No. 44	2013.10.25	200	japonica	C
Zhendao No. 10	2013.10.25	200	japonica	D
Xinliangyou No. 6380	2013.10.15	200	indica	E
Liangyou No. 0293	2013.10.15	200	indica	F

**Table 2:** Statistics regarding the estimation error of the spikelet number per panicle.

Panicle varieties	Error range/%	20% test			Calibration curves test	
		Area/%	TLPB2(image)/%	TLPB2/%(manual)	Area/%	TLPB2/%
A	5	88.89	55.56	57.89	82.76	54.02
	10	100	72.22	84.21	100	80.46
B	5	82.86	68.57	71.43	84.80	67.25
	10	100	100	95.24	98.25	93.57
C	5	59.52	42.86	78.05	72.08	51.27
	10	97.62	88.10	90.24	97.46	82.23
D	5	60.61	39.39	57.50	69.23	40.66
	10	96.97	78.79	87.50	93.96	73.08
E	5	57.50	35	48.78	65.46	34.54
	10	90	65	68.29	93.30	61.34
F	5	82.50	60	60.98	84.54	57.22
	10	100	90	85.37	98.45	85.05

Error range: sample number (error less than 5% or 10%) divided by the total number of examined samples for each variety.

**Table 3:** Estimation of the deviation of the spikelet number per square.

Panicle varieties	TRUE	Area	20% test	TRUE	Calibration curves test			
			TLPB2		TLPB2	TRUE	Area/%	TLPB2/%
			(image)		(manual)			
A	93.3	92.4	93.3	96.1	96.8	88.7	88.7	90.2
		−0.96%	0		0.73%		0	1.69%
B	116.5	115.5	116.3	111.0	110.7	108.8	110.3	110.0
		0.86%	0.17%		0.27%		1.38%	1.10%
C	105.9	107.1	105.6	106.1	106.4	112.9	112.9	112.8
		1.13%	0.28%		0.28%		0	−0.09%
D	90.0	89.6	90.4	99.2	99.0	89.4	90.6	89.9
		−0.44%	0.44%		−0.20%		1.34%	0.56%
E	185.2	185.0	186.8	207.2	212.8	182.3	184.2	186.1
		−0.11%	0.86%		2.70%		1.04%	2.08%
F	203.2	203.0	198.2	197.2	204.0	206.4	206.8	206.9
		−0.10%	−2.46%		3.45%		0.19%	0.24%

TRUE: The spikelet number per panicle was counted manually and averaged. The other values were estimated and averaged using the corresponding linear regression equations.
